# Interleukin-8 promotes integrin β3 upregulation and cell invasion through PI3K/Akt pathway in hepatocellular carcinoma

**DOI:** 10.1186/s13046-019-1455-x

**Published:** 2019-11-04

**Authors:** Fengkai Sun, Jianping Wang, Qi Sun, Fanni Li, Hengjun Gao, Lin Xu, Jiao Zhang, Xiaoyan Sun, Yanan Tian, Qiujie Zhao, Huimin Shen, Kai Zhang, Jun Liu

**Affiliations:** 1 Department of Gastroenterology, Shandong Provincial Hospital Affiliated to Shandong University, Jingwu Road 324#, Jinan, 250021 China; 2 Department of Hepatobiliary Surgery, Shandong Provincial Hospital Affiliated to Shandong University, Jinan, 250021 Shandong China; 3 Department of General Surgery, The First Affiliated Hospital of Xi’an Jiaotong University, Xi’an, 710061 Shaanxi China; 4 Department of Talent Highland, The First Affiliated Hospital of Xi’an Jiaotong University, Xi’an, 710061 Shaanxi China

**Keywords:** Interleukin-8, Integrin αvβ3, Invasion, Hepatocellular carcinoma

## Abstract

**Background:**

Interleukin-8 (IL-8) plays a vital role in the invasion and metastasis of hepatocellular carcinoma (HCC), and is closely associated with poor prognosis of HCC patients. Integrin αvβ3, a member of the integrin family, has been reported to be overexpressed in cancer tissues and mediate the invasion and metastasis of HCC cells. However, the relationship between IL-8 and integrin αvβ3 in HCC and the underlying mechanism of IL-8 and integrin αvβ3 in the invasion of HCC remains unclear.

**Methods:**

The expression of IL-8, integrin αv and integrin β3 in HCC cells and tissues was detected by quantitative real-time PCR, Western blot and immunohistochemistry. Transwell assay and Western blot was used to detect the invasiveness, the expression of integrin β3 and the activation of PI3K/Akt pathway of HCC cells pretreated with IL-8 knockdown or exogenous IL-8.

**Results:**

IL-8, integrin αv and integrin β3 were overexpressed in highly metastatic HCC cell lines compared with low metastatic cell lines. There was a positive correlation between integrin β3 and IL-8 expression in HCC tissues. IL-8 siRNA transfection reduced HCC cell invasion and the levels of integrin β3, p-PI3K and p-Akt. IL-8 induced HCC cell invasion and integrin β3 expression was significantly inhibited by transfection with CXCR1 siRNA or CXCR2 siRNA. When we stimulated HCC cells with exogenous IL-8, cell invasion and the levels of integrin β3, p-PI3K, and p-Akt increased, which could be effectively reversed by adding PI3K inhibitor LY294002.

**Conclusions:**

Our results suggest that IL-8 promotes integrin β3 upregulation and the invasion of HCC cells through activation of the PI3K/Akt pathway. The IL-8/CXCR1/CXCR2/PI3K/Akt/integrin β3 axis may serve as a potential treatment target for patients with HCC.

## Background

Although much progress has been made in diagnosis and treatment in recent years, hepatocellular carcinoma (HCC) remains one of the leading causes of cancer-related death worldwide [1]. Tumor invasion and metastasis are important factors leading to poor clinical outcome in patients with HCC. A better understanding of the molecular mechanism underlying tumor invasion and metastasis is of great significance for improving the prognosis of HCC.

Inflammatory tumor microenvironment is crucial in the development and metastasis of HCC [2]. Proinflammatory cytokines secreted in tumor microenvironment play a key role in the initiation, growth, progression, and metastasis of various tumors [3]. Interleukin-8 (IL-8), also known as CXCL8, which is secreted by tumor and inflammatory cells, promotes tumor migration, invasion, angiogenesis, and metastasis by binding with its receptors, CXCR1 and CXCR2 [4–6]. Overexpression of IL-8 is closely related to vascular invasion, lymphatic, and intrahepatic metastasis, advanced tumor stage, and early tumor recurrence, and can predict the adverse clinical prognosis of various tumors, including HCC [7, 8].

Invasion and metastasis of cancer cells is a complex, dynamic and multistage process, involving a series of changes in the interaction between invasive cells and extracellular matrix or neighboring cells. Integrins represent a large family of cell-surface receptors which not only play an important role in normal physiological development, but also regulate a variety of cellular functions essential for cancer progression [9]. Integrins are transmembrane heterodimeric molecules composed of α and β subunits. Due to various combinations of α and β subunits, at least 24 distinct integrins have been described. Integrin αvβ3, which has been detected in various types of tumors including carcinoma of pancreas, prostate, breast, and cervix, plays a critical role in tumor cell proliferation, migration, invasion, metastasis, and angiogenesis. The expression of integrin αvβ3 closely associates with disease progression and prognosis in a variety of tumors [10–12]. As respect to HCC, integrin αvβ3 has also been reported to be overexpressed in carcinoma tissue and mediate the invasion and metastasis of HCC cells [13–16].

IL-8, rarely detectable in physiological states, is rapidly induced after stimulation by proinflammatory factors under pathological conditions where integrin αvβ3 is also upregulated [16]. Both IL-8 and integrin αvβ3 have been reported to play vital roles in HCC cell invasion [6–8, 13–16]. However, the association between IL-8 and integrin αvβ3 in HCC and the underlying mechanism of IL-8 and integrin αvβ3 in HCC invasion remains largely unknown. In this study, we explored the effect of IL-8 on the expression of integrin αvβ3 and the mechanism of IL-8 regulating the expression of integrin αvβ3 and promoting the invasion of HCC cells.

## Materials and methods

### Cell culture and transfection

The HCC cell lines HCCLM3 and MHCC97H were established in the Liver Cancer Institute of the Zhongshan Hospital of Fudan University (Shanghai, China). Huh-7 and HepG2 were purchased from Procell (Wuhan, China). All cell lines were cultured in DMEM supplemented with 10% fetal bovine serum and penicillin-streptomycin antibiotic mixture (10 ml/L; HyClone) at 37 °C in an atmosphere of 5% CO_2_. PI3K inhibitor LY294002 (25 μM, Selleck) was used to inhibit the activation of PI3K. Human IL-8 siRNA (#sc-156,051), integrin β3 siRNA (#sc-29,375), and control siRNA (#sc-37,007) were purchased from Santa Cruz. CXCR1 siRNA and CXCR2 siRNA were synthesized by GenePharma Co., Ltd. (Shanghai, China) [17]. Plasmid encoding integrin β3 and empty vector control plasmid were purchased from GeneChem Co., Ltd. (Shanghai, China). HCC cells were seeded in six-well plates, incubated overnight. Transfection of siRNA or plasmids was performed using Lipofectamine 3000 (Invitrogen).

### Clinical samples

The study protocol was performed following the Declaration of Helsinki. One hundred and thirty-six HCC patients who underwent surgery in Shandong Provincial Hospital affiliated to Shandong University were enrolled from 2013 to 2018. Samples were collected after approval by the Medical Ethics Committee of Shandong Provincial Hospital affiliated to Shandong University and written informed consents were obtained from each patient. Histopathologic sections were diagnosed by two pathologists.

### Immunohistochemistry

After routine deparaffinization, rehydration, and antigen retrieval, the sections were incubated with monoclonal antibodies against IL-8 (1:2000, #ab18672, Abcam), integrin αv (1:100; # sc-9969, Santa Cruz), or integrin β3 (1:100, #sc-52,589, Santa Cruz) at 4 °C overnight, followed by incubation with the corresponding secondary antibodies at 37 °C for half an hour and visualization using DAB.

Five representative fields (200× magnification) for each section were randomly selected for histological analysis to evaluate the expression of IL-8, integrin αv and integrin β3. In order to quantify the gene expression level, we used a score based on two parameters: staining intensity and staining area. The staining intensity was scored as 0 (negative), 1 (weak), 2 (moderate), or 3 (strong). The staining area was scored as 0 (0%), 1 (≤5%), 2 (5–50%), or 3 (≥50%). A final score < 4 was defined as negative expression, and a score ≥ 4 was defined as positive expression.

### Transwell assay

Cell invasion and migration was measured using 24-well Transwell chamber (Corning Costar) precoated with or without Matrigel. Cells in serum-free medium were seeded at 1 × 10^5^ per well in the top chamber, while 15% FBS in DMEM medium was added in the bottom chamber. After incubation for 24 h at 37 °C, cells were fixed with methanol, stained with 0.1% crystal violet or 0.4% trypan blue and counted.

### Western blot analysis

Equivalent amounts of proteins were subjected to SDS-PAGE gel separation, followed by transfer onto PVDF membranes, and then blocked for 1 h in 5% skim milk diluted with TBST and incubated overnight at 4 °C with corresponding primary antibodies. After three rinses with TBST, the membranes were incubated with HRP-conjugated secondary antibody for 1 h at room temperature, and finally exposed using enhanced chemiluminescence. Primary antibodies were listed as below: integrin αv (1:700; # sc-9969, Santa Cruz), integrin β3 (1:700, #sc-52,589, Santa Cruz), IL-8 (1:2000, #ab18672, Abcam), phospho-PI3K p85 (1:600, #4228, CST), PI3K p85 (1:600, #4257, CST), phosphor-Akt (1:600, #4060, CST), and Akt (1:2000, #4691, CST).

### Quantitative real-time PCR

Total RNA was extracted using Trizol reagent (Invitrogen). cDNAs were synthesized using Revert Aid™ First Strand cDNA Synthesis Kit (Fermentas). Quantitative real-time PCR was performed on the Light Cycler 480 (Roche Applied Science) by using SYBR® Premix Ex TaqTM II (TaKaRa). Glyceraldehyde-3-phosphate dehydrogenase (GAPDH) was used as the internal control. The sequences are described as follows: integrin αv 5′-CCAGGTGGTATGTGACCTTGGA-3′ (forward) and 5′-GCTGGTGCACACTGAAACGAA-3′ (reverse); integrin β3 5′-TTCAATGCCACCTGCCTCAA-3′ (forward) and 5′-TTGGCCTCAATGCTGAAGCTC-3′ (reverse); IL-8 5′-ACACTGCGCCAACACAGAAATTA-3′ (forward) and 5′-TTTGCTTGAAGTTTCACTGGCATC-3′ (reverse); GAPDH 5′-GCACCGTCAAGGCTGAGAAC-3′ (forward) and 5′-TGGTGAAGACGCCAGTGGA-3′ (reverse). Quantitative values were obtained by the threshold cycle (Ct) value, which was normalized using the mean Ct for the reference gene, GADPH. The 2^−ΔΔCt^ method was used to perform the data analyses.

### Statistical analysis

Statistical analyses were conducted using SPSS 22.0 software. The quantitative variables were compared using Student’s t-test or Mann-Whitney U-test. The categorical variables were analyzed by Chi-square test. Spearman’s rank correlation test was used for correlation analysis between the expression of IL-8 and integrin αv or β3. Statistically significant was considered as *P* < 0.05.

## Results

### IL-8, integrin αv and integrin β3 were overexpressed in highly metastatic HCC cell lines

**Fig. 1:**
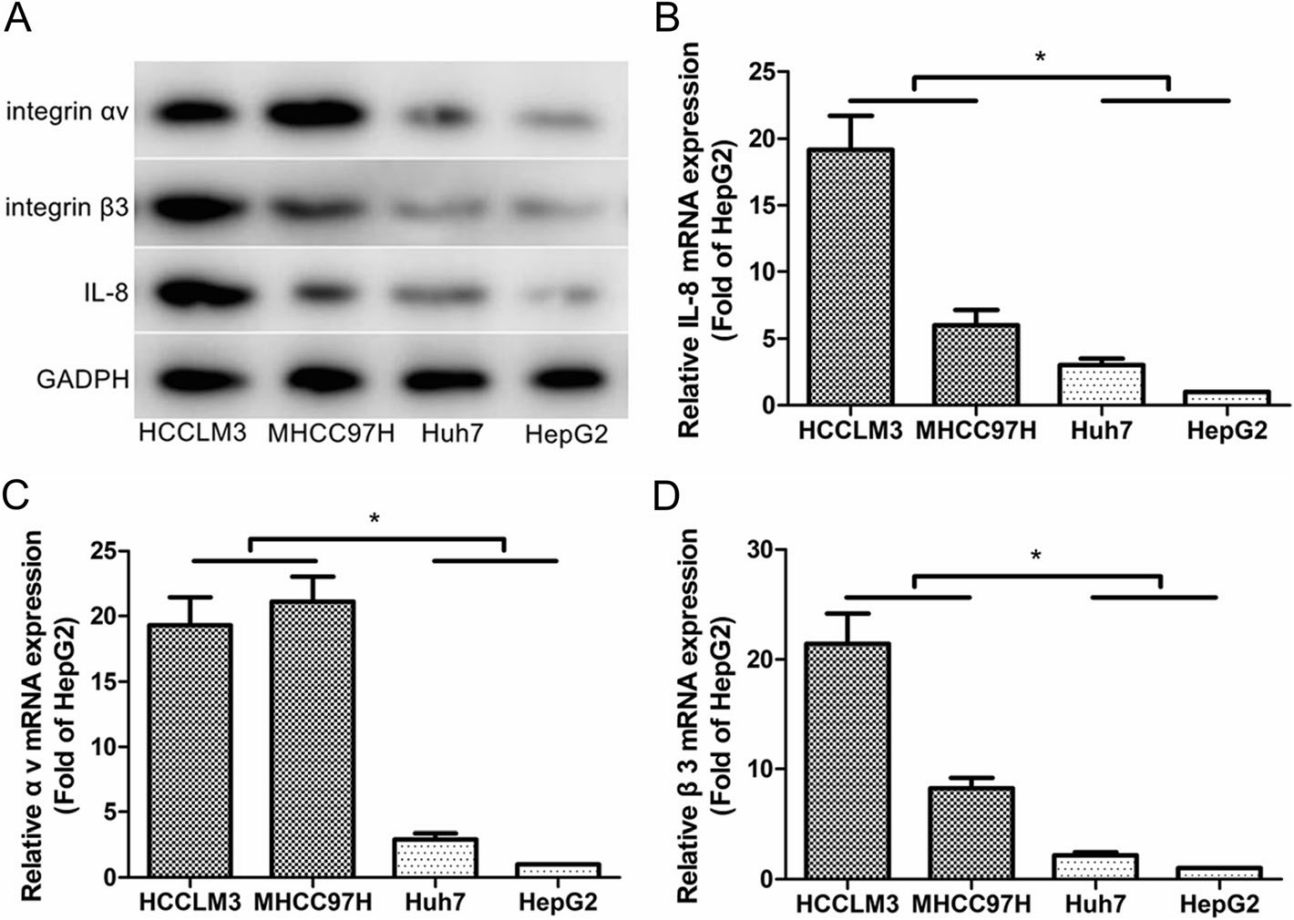
IL-8 and integrin αvβ3 were overexpressed in highly metastatic HCC cell lines (HCCLM3 and MHCC97H) compared with low metastatic cell lines (Huh-7 and HepG2). **a** Western blot analysis of IL-8, integrin αv and β3 protein in the indicated cells. **b** Real-time PCR analysis of IL-8 expression in the indicated cells. **c** Real-time PCR analysis of integrin αv expression in the indicated cells. **d** Real-time PCR analysis of integrin β3 expression in the indicated cells. Results are expressed as the mean ± SEM. *P < 0.05

To investigate the role of IL-8, integrin αv and integrin β3 in HCC metastasis, we detected the expression of IL-8, integrin αv and integrin β3 in several HCC cell lines with different metastatic potential. Western blot and real-time PCR analysis confirmed that both protein expression and mRNA levels of IL-8, integrin αv and integrin β3 were significantly increased in highly metastatic HCC cell lines (HCCLM3 and MHCC97H) compared with low metastatic cell lines (Huh-7 and HepG2; Fig. 1).

### There was a positive correlation between integrin β3 and IL-8 expression in HCC tissues

**Fig. 2:**
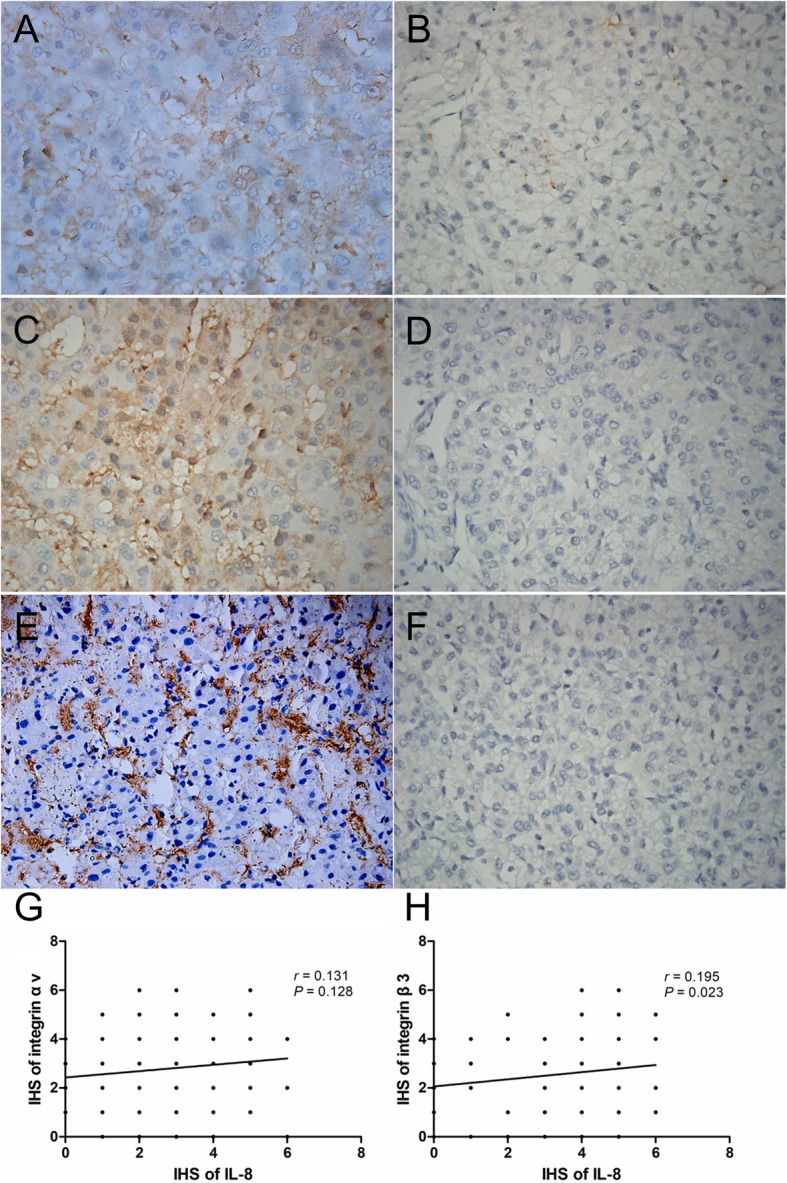
Immunohistochemical staining of IL-8, integrin αv and integrin β3 in HCC (original magnification, × 400). **a** High IL-8 expression. **b** Low IL-8 expression. **c** Positive integrin αv expression. **d** Negative integrin αv expression. **e** Positive integrin β3 expression. **f** Negative integrin β3 expression. **g** Correlation analysis between the expression of IL-8 and integrin αv. **h** Correlation analysis between the expression of IL-8 and integrin β3

The expression of IL-8, integrin αv and integrin β3 was examined in a cohort of 136 HCC patients using immunohistochemistry. The results suggested that IL-8 staining was mainly distributed in the cytoplasm, while integrin αv and β3 subunit staining was localized to the plasma membrane (Fig. 2a-f). We found high IL-8 expression in 70 cases (51.5%), positive integrin αv expression in 64 cases (47.1%) and positive integrin β3 expression in 61 cases (44.9%), respectively. We further explored the association between IL-8 expression and integrin αv or integrin β3 expression in 136 HCC samples. Immunohistochemical analysis showed that IL-8 expression was positively correlated with integrin β3 expression (r = 0.195, *P* = 0.023, Fig. 2h). However, no correlation was found between the expression of IL-8 and integrin αv (r = 0.131, *P* = 0.128, Fig. 2g).

### IL-8 knockdown decreased the expression of integrin β3 and the invasion and migration of HCC cells

**Fig. 3:**
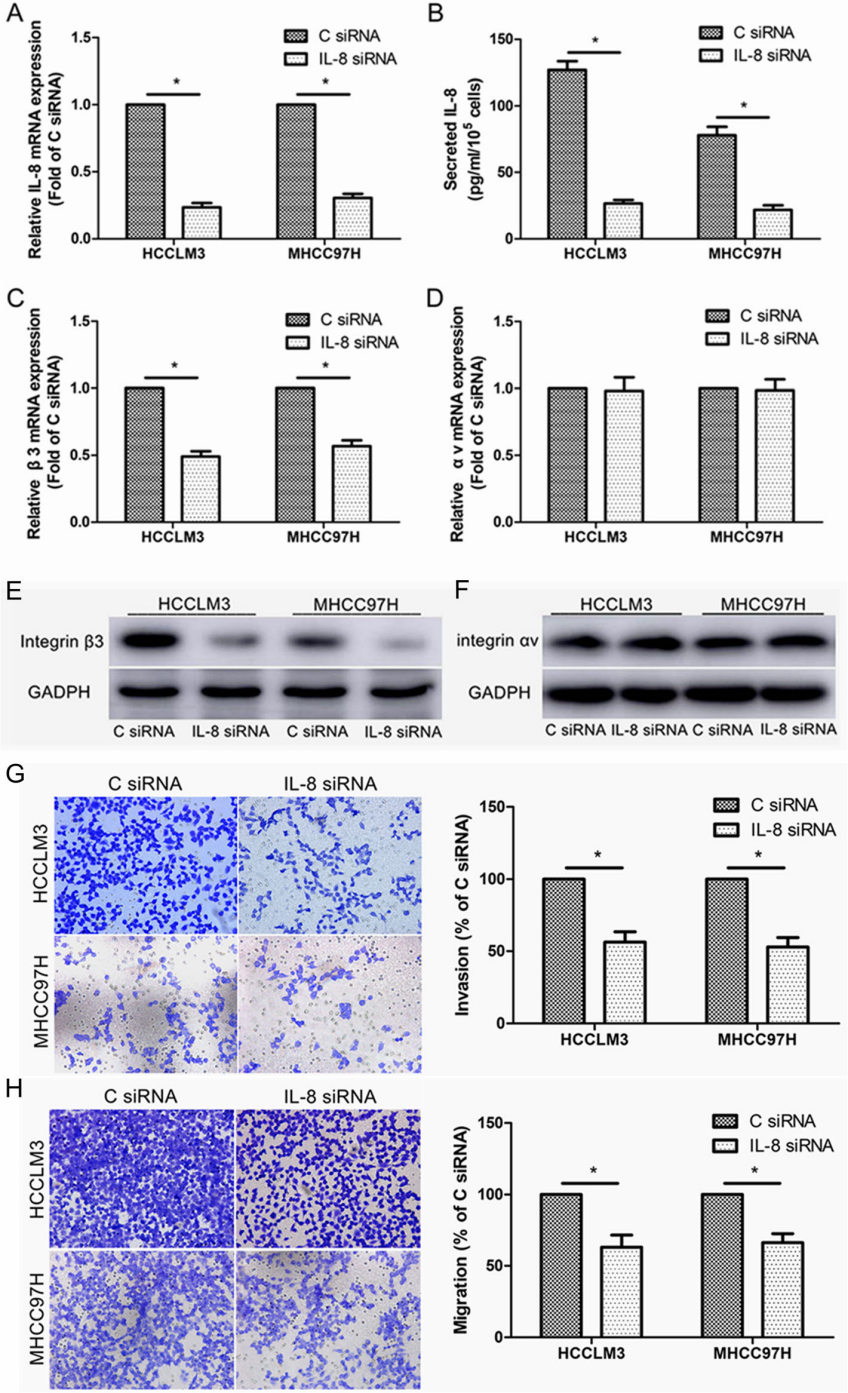
IL-8 knockdown decreased integrin β3 expression and HCC cell invasion and migration. **a** Real-time PCR analysis of IL-8 mRNA level after 48 h of transfection with IL-8 siRNA or control siRNA (C siRNA). **b** ELISA analysis of IL-8 protein level after 48 h of transfection with IL-8 siRNA or C siRNA. **c** Real-time PCR analysis of integrin β3 mRNA level after 48 h of transfection with IL-8 siRNA or C siRNA. **d** Real-time PCR analysis of integrin αv mRNA level after 48 h of transfection with IL-8 siRNA or C siRNA. **e** Western blot analysis of integrin β3 expression after 48 h of transfection with IL-8 siRNA or C siRNA. **f** Western blot analysis of integrin αv expression after 48 h of transfection with IL-8 siRNA or C siRNA. **g** Invasion assay of HCCLM3 and MHCC97H cells after 48 h of transfection with IL-8 siRNA or C siRNA. **h** Migration assay of HCCLM3 and MHCC97H cells after 48 h of transfection with IL-8 siRNA or C siRNA. *P < 0.05 compared to cells transfected with C siRNA

To further investigate whether IL-8 can regulate the expression of integrin αvβ3 in HCC cells, we transfected HCCLM3 and MHCC97H cells with IL-8 siRNA, which resulted in a significant decrease in the level of IL-8 mRNA and the level of IL-8 secreted by HCC cells, suggesting the effective knockdown of IL-8 by IL-8 siRNA (Fig. 3a, b). The results showed that knocking down IL-8 decreased the expression of integrin β3 at the level of mRNA and protein in HCCLM3 and MHCC97H cells (Fig. 3c, e). However, knockdown of IL-8 had no significant effect on integrin αv expression in HCC cells (Fig. 3d, f). Besides, we found that transfection of IL-8 siRNA significantly reduced the invasion and migration of HCCLM3 and MHCC97H cells, compared to control siRNA (Fig. 3g, h).

### IL-8 induced HCC cell invasion through CXCR1/2 receptors

**Fig. 4:**
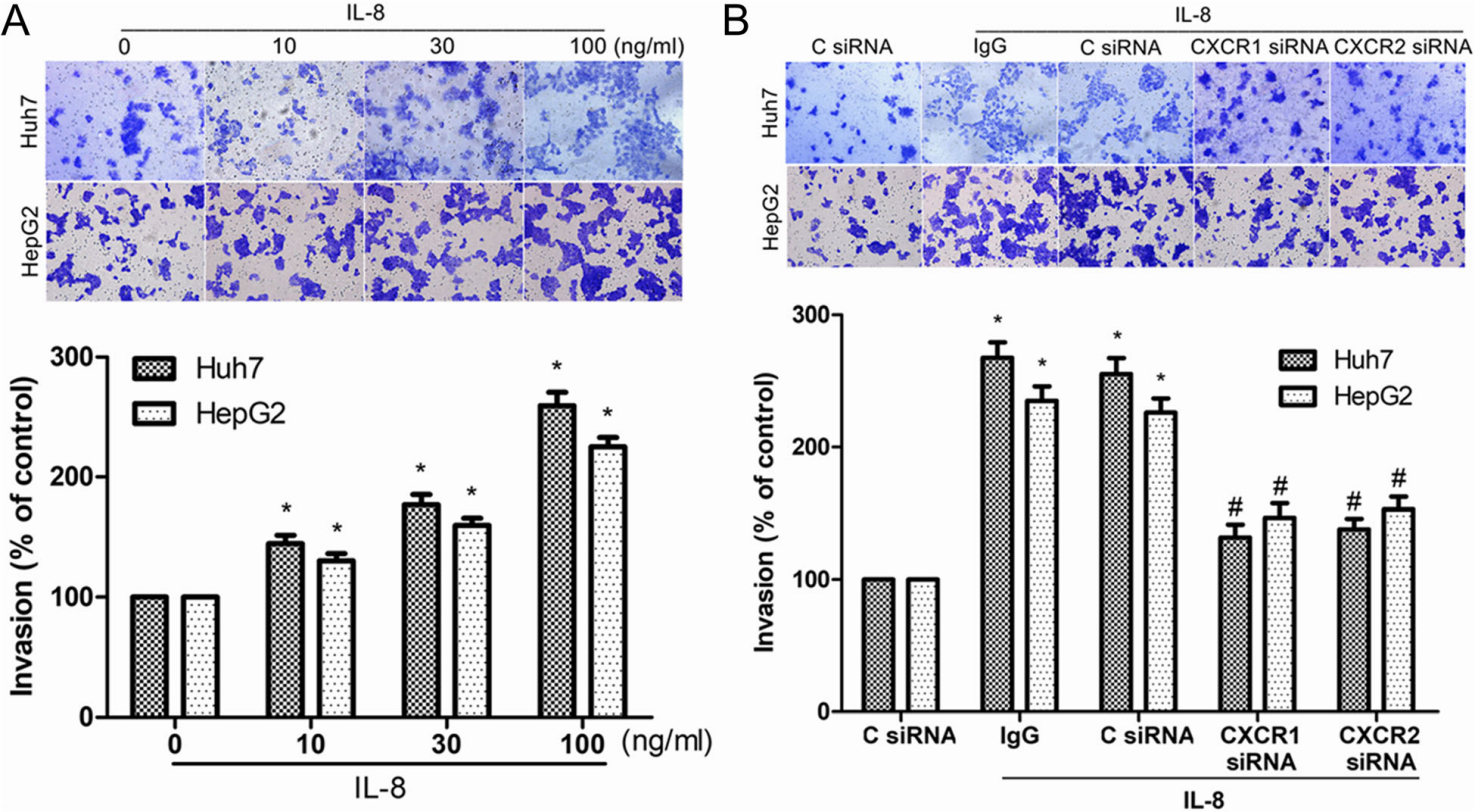
IL-8 increased HCC cell invasion through CXCR1/2 receptors. **a** Huh-7 and HepG2 cells were incubated with various concentrations of IL-8 for 24 h, and in vitro invasion was measured with Transwell assay. **b** After 48 h of transfection with CXCR1 siRNA, CXCR2 siRNA or control siRNA (C siRNA), cells were incubated with IL-8 (100 ng/ml) for 24 h, and in vitro invasion was measured with Transwell assay. Results are expressed as the mean ± SEM. *P < 0.05 compared with the control group. #*P* < 0.05 compared with the IL-8-treated group

It has been reported that IL-8 can promote the invasion of various cancer cells including HCC cells [8]. Next we investigated the effects of various concentrations of IL-8 on the invasion of Huh7 and HepG2 cells. The results showed that IL-8 increased the invasion of Huh7 and HepG2 cells in a concentration-dependent manner (Fig. 4a). IL-8 has been reported to induce cancer cell invasion by interacting with its receptors, CXCR1 and CXCR2, on the surface of cancer cells [4–6]. In this study, IL-8-mediated HCC cell invasion was significantly inhibited by transfection with CXCR1 siRNA or CXCR2 siRNA (Fig. 4b). Our study provided evidence that IL-8 probably induced invasion of HCC cells through CXCR1 and CXCR2.

### IL-8 increased HCC cell invasion by upregulating the expression of integrin β3

**Fig. 5:**
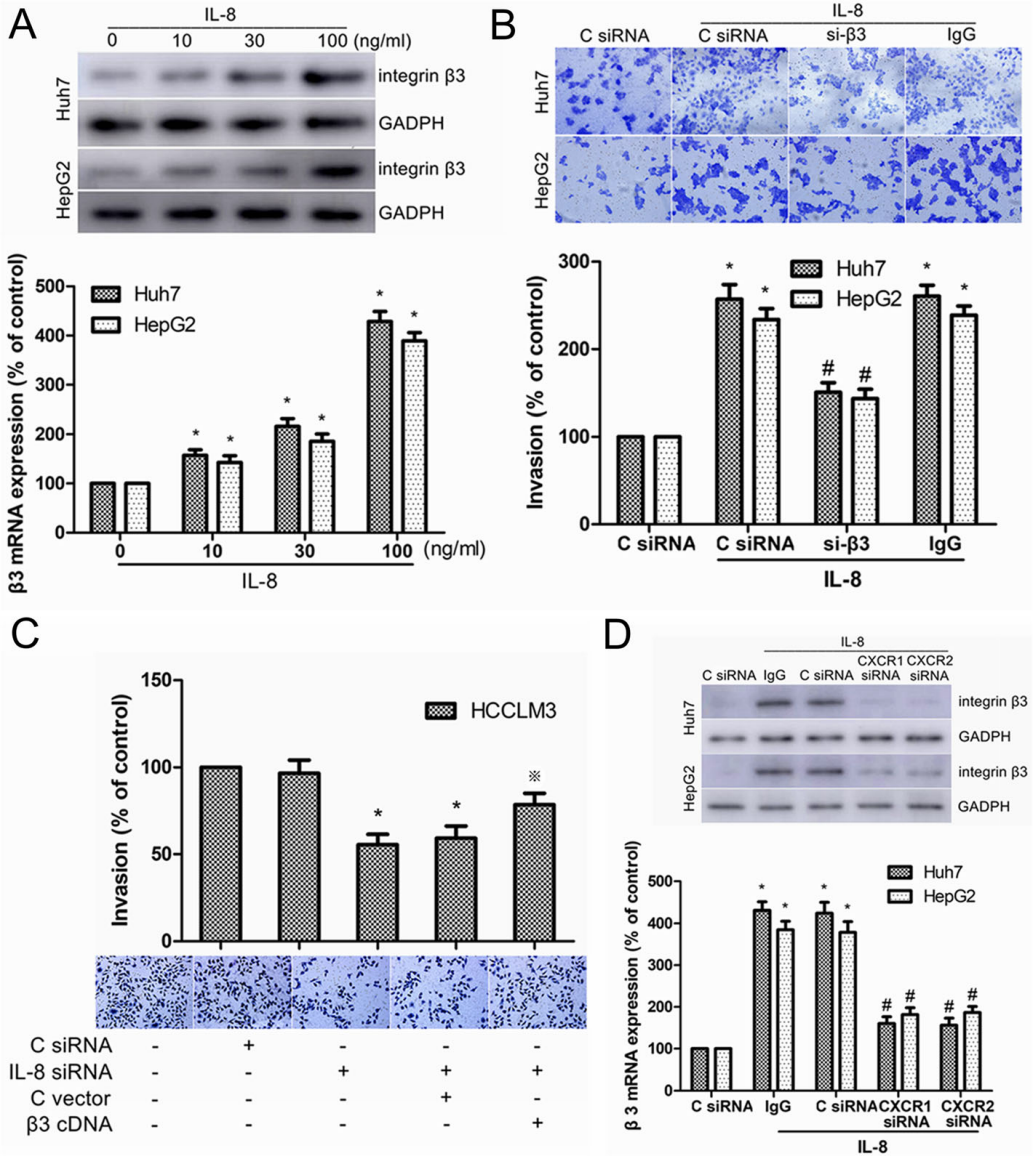
Integrin β3 was involved in IL-8/CXCR1/2-induced invasion of HCC cells. **a** Real-time PCR and Western blot analysis of integrin β3 expression in Huh-7 and HepG2 cells after incubation with different concentrations of IL-8 for 24 h. **b** Huh-7 and HepG2 cells were transfected with integrin β3 siRNA (si-β3) or control siRNA (C siRNA) for 48 h, and in vitro invasion was measured with Transwell assay in the presence or absence of IL-8 (100 ng/ml) for 24 h. **c** HCCLM3 cells were transfected using IL-8 siRNA with or without integrin β3 cDNA, and in vitro invasion was measured with Transwell assay. **d** Real-time PCR and Western blot analysis of integrin β3 expression in Huh-7 and HepG2 cells after transfection of CXCR1 siRNA, CXCR2 siRNA or C siRNA followed by stimulation with IL-8 (100 ng/ml) for 24 h. Results are expressed as the mean ± SEM. *P < 0.05 compared with the control group. #*P* < 0.05 compared with the IL-8-treated group. ※*P* < 0.05 compared with the IL-8 siRNA-treated group

Previous studies indicated that integrin αvβ3 participated in the invasion and metastasis of HCC cells [13–15]. In this study, we found IL-8 knockdown reduced integrin β3 expression and invasion of HCC cells. Therefore, we speculated that integrin β3 might participate in IL-8-mediated invasion of HCC cells. In Huh7 and HepG2 cells, IL-8 was found to increase mRNA level and protein expression of integrin β3 in a dose-dependent manner (Fig. 5a). Moreover, transfection of integrin β3 siRNA (si-β3) significantly suppressed IL-8-induced cell invasion (Fig. 5b). IL-8 siRNA-induced reduction in HCCLM3 cell invasion could be rescued by overexpression of integrin β3 (Fig. 5c). Besides, transfection of CXCR1/2 siRNA reduced the transcription and translation of integrin β3 induced by IL-8 (Fig. 5d). In summary, our results indicated that IL-8/CXCR1/2 increased HCC cell invasion by upregulating the expression of integrin β3.

### PI3K/Akt signaling pathway participated in IL-8 mediated HCC cell invasion and integrin β3 upregulation

**Fig. 6:**
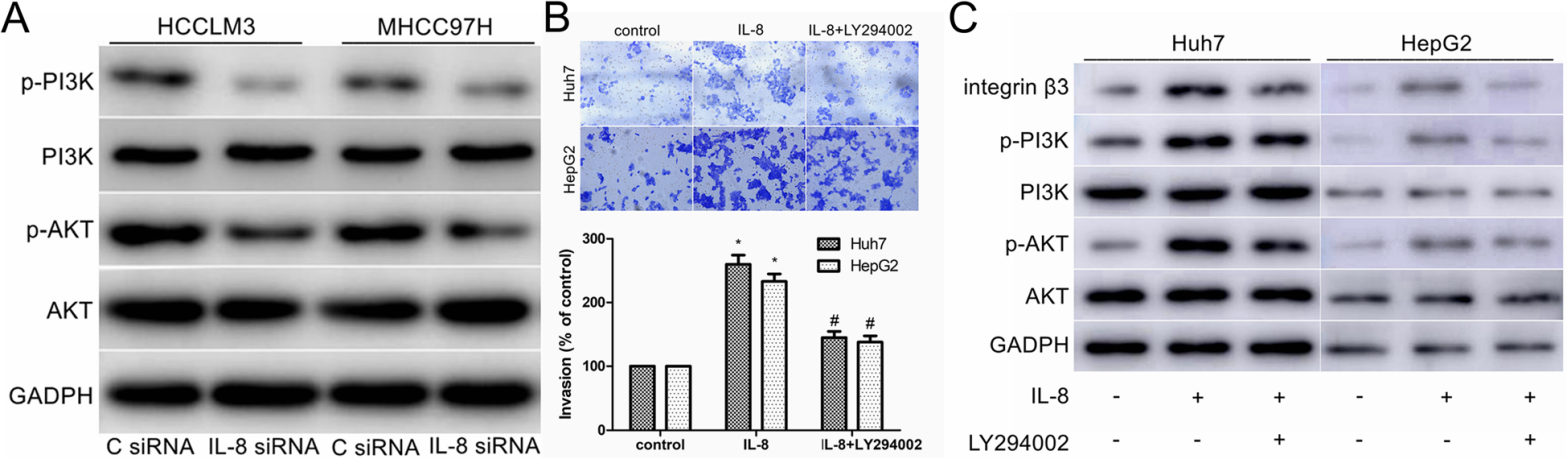
PI3K/Akt signaling pathway was involved in IL-8 mediated HCC cell invasion and integrin β3 upregulation. **a** Western blot analysis of total and phosphorylated PI3K and Akt in C siRNA and IL-8 siRNA transfected cells. **b** After pretreatment with PI3K-specific inhibitor LY294002 (25 μM) for 30 min, cells were stimulated with IL-8 (100 ng/ml) for 24 h, and in vitro invasion was measured with Transwell assay. **c** After pretreatment with LY294002 (25 μM) for 30 min, cells were stimulated with IL-8 (100 ng/ml) for 24 h, and expression of integrin β3, PI3K, p-PI3K, Akt and p-Akt was measured by western blot. Results are expressed as the mean ± SEM. *P < 0.05 compared with the control group. #*P* < 0.05 compared with the IL-8-treated group

It has been reported that PI3K/Akt signaling pathway mediated the migration and invasion of cancer cells by upregulating the expression of integrin β3 [18, 19]. In this study, we have proved that IL-8 siRNA transfection significantly reduced the expression of integrin β3 and the invasion of HCCLM3 and MHCC97H cells (Fig. 3c, e, g). To further investigate whether the PI3K/Akt pathway was involved in IL-8 mediated cell invasion and integrin β3 upregulation, we transfected HCCLM3 and MHCC97H cells with IL-8 siRNA and found that the levels of total PI3K and total Akt were not changed but the levels of phosphorylated PI3K (p-PI3K) and phosphorylated Akt (p-Akt) were reduced (Fig. 6a).

In addition, the cell invasion and the levels of p-PI3K and p-Akt increased when Huh7 and HepG2 cells were stimulated with IL-8 (Fig. 6b, c). Pretreatment of Huh7 and HepG2 cells with PI3K inhibitor LY294002 reduced IL-8 mediated cell invasion (Fig. 6b). Besides, addition of LY294002 also effectively reversed the upregulation of integrin β3 induced by IL-8 and decreased the phosphorylation of PI3K/Akt (Fig. 6c). Our study provided evidence that PI3K/Akt signaling pathway participated in IL-8-mediated HCC cell invasion and integrin β3 upregulation.

## Discussion

IL-8 is an important multifunctional cytokine that not only participates in various inflammatory diseases, but also mediates tumor migration, invasion and angiogenesis by binding CXCR1 and CXCR2 [4, 20]. Previous studies have reported that IL-8 overexpression is closely related to aggressive behavior of tumors such as vascular invasion, lymphatic and intrahepatic metastasis, advanced tumor stage, and early tumor recurrence, and can predict the adverse clinical prognosis of HCC [7, 8]. Integrin αvβ3, as an important cell surface adhesion molecule, has also been reported to be overexpressed in cancer tissues and mediate the invasion and metastasis of HCC cells [13–16]. Increasing evidence suggests that the close relationship between IL-8 and integrin is critical for cancer progression [18, 19, 21, 22]. To explore the role of IL-8 and integrin αvβ3 in HCC metastasis, we detected the expression of IL-8, integrin αv and integrin β3 in several HCC cell lines with different metastatic potential, and found that both protein expression and mRNA levels of IL-8, integrin αv, and integrin β3 were significantly increased in highly metastatic HCC cell lines (HCCLM3 and MHCC97H) compared with low metastatic cell lines (Huh-7 and HepG2). We further analyzed the expression of IL-8, integrin αv and β3 in 136 HCC specimens, and found that there was a positive correlation between the expression of IL-8 and integrin β3. In order to further investigate whether endogenous IL-8 regulates the expression of integrin β3 in HCC cells, we transfected HCCLM3 and MHCC97H cells with IL-8 siRNA, which led to a significant decrease in the expression of integrin β3 and reduced the invasion and migration of HCCLM3 and MHCC97H cells. Moreover, exogenous IL-8 was found to dose-dependently increase mRNA level and protein expression of integrin β3 in Huh7 and HepG2 cells. Inhibition of integrin β3 by pretreatment with si-β3 significantly reduced IL-8-induced cell invasion, while IL-8 siRNA-induced reduction in HCCLM3 cell invasion could be rescued by overexpression of integrin β3. All these data suggest that IL-8 increases the invasion of HCC cell by upregulating the expression of integrin β3.

IL-8 and its receptors, CXCR1 and CXCR2, have been extensively reported to mediate invasion, angiogenesis, and metastasis of various cancers [4, 20]. Huang et al. reported that downregulation of CXCR1 dramatically reduced HCC cell migration, invasion in vitro and lung metastasis in mice model, and HCC patients with positive expression of IL-8 or CXCR1 had shorter overall survival time and higher recurrence rate compared with those with negative expression [6]. Wu et al. reported that inhibition of CXCR2 by siRNA dramatically decreased HCC cell migration in vitro [23]. Li et al. demonstrated that an antagonist for CXCR1 and CXCR2 inhibited the proliferation of HCC cells in vitro and cancer development in vivo [24]. Consistent with the above studies, IL-8 was found to induce HCC cell invasion through CXCR1/2 receptors in our study. Moreover, CXCR1 and CXCR2 were found to be involved in IL-8-mediated integrin β3 upregulation, which could be reversed by transfection with CXCR1/2 siRNA.

PI3K/Akt pathway is a major downstream signaling pathway of IL-8 inducing cancer cell migration, invasion, and metastasis [6, 18, 19, 25, 26]. Moreover, PI3K/Akt has been reported to mediate the migration and invasion of cancer cells by upregulating the expression of integrin β3 [18, 19]. In this study, we confirmed that knocking down IL-8 resulted in significant decrease in integrin β3, p-PI3K, and p-Akt levels and reduced the invasion of HCCLM3 and MHCC97H cells, suggesting that integrin β3 and PI3K/Akt were involved in IL-8-mediated invasion of HCC cells. When we stimulated Huh7 and HepG2 cells with exogenous IL-8, the levels of cell invasion and integrin β3, p-PI3K, and p-Akt increased, which could be effectively reversed by adding PI3K inhibitor LY294002. In conclusion, our results indicated that PI3K/Akt pathway was involved in IL-8-mediated invasion of HCC cells and upregulation of integrin β3.

## Conclusions

In summary, we revealed a positive correlation between integrin β3 and IL-8 expression in HCC tissue and the vital role of integrin β3 in IL-8 mediated invasion of HCC cells. Our results suggest that IL-8 promotes the upregulation of integrin β3 and the invasion of HCC cells through activating the PI3K/Akt signaling pathway. This provides evidence that the IL-8/CXCR1/CXCR2/PI3K/Akt/integrin β3 axis may serve as a potential treatment target for patients with HCC.

## Data Availability

All data generated or analyzed during the present study are included in this published article.

## Abbreviations

CXCR1: C-X-C chemokine receptor 1
CXCR2: C-X-C chemokine receptor 2
GAPDH: Glyceraldehyde-3-phosphate dehydrogenase
HCC: Hepatocellular carcinoma
IL-8: Interleukin-8
p-Akt: Phosphorylated Akt
p-PI3K: Phosphorylated PI3K
